# Supplementary material for: Employing Gamma-Ray-Modified Carbon Quantum Dots to Combat a Wide Range of Bacteria
Source: Antibiotics (Basel). 2023 May 17;12(5):919. doi: 10.3390/antibiotics12050919 (PMC10215299; doi:10.3390/antibiotics12050919)
Supplement: Supplementary file 1 [file antibiotics-12-00919-s001.zip › antibiotics-2389786-supplementary.pdf]

Supporting information

Employing gamma rays modified carbon quantum dots to combat wide range of bacteria

Zoran M. Marković[1], Aleksandra S. Mišović[2], Danica Z. Zmejkoski[1], Nemanja M. Zdravković[2], Janez Kovač[3], Danica V. Bajuk-Bogdanović[4], Dušan D. Milivojević[1], Marija M. Mojsin[5], Milena J. Stevanović[5,6,7], Vladimir B. Pavlović[8], Biljana M. Todorović Marković[1]*

[1]Vinča Institute of Nuclear Sciences, National Institute of the Republic of Serbia, University of Belgrade, Mike Alasa 12-14, 11001 Belgrade, Serbia

[2]Faculty of Physical Chemistry, University of Belgrade, Studentski trg 12-16, 11158 Belgrade, Serbia

[3]Scientific Veterinary Institute of Serbia, Janisa Janulisa 14, 11107 Belgrade, Serbia

[4]Jozef Stefan Institute, Department of Surface Engineering - F4, Jamova cesta 39, 1000Ljubljana, Slovenia

[5]Institute of Molecular Genetics and Genetic Engineering, University of Belgrade, Vojvode Stepe 444a, 11042 Belgrade, Serbia

[6]Faculty of Biology, University of Belgrade, Studentski trg 16, 11000 Belgrade, Serbia

[7]Serbian Academy of Sciences and Arts, Knez Mihailova 35, 11000 Belgrade, Serbia

[8]Faculty of Agriculture, University of Belgrade, Nemanjina 6, 11080 Belgrade-Zemun, Serbia

*corresponding author. email: biljatod@vin.bg.ac.rs

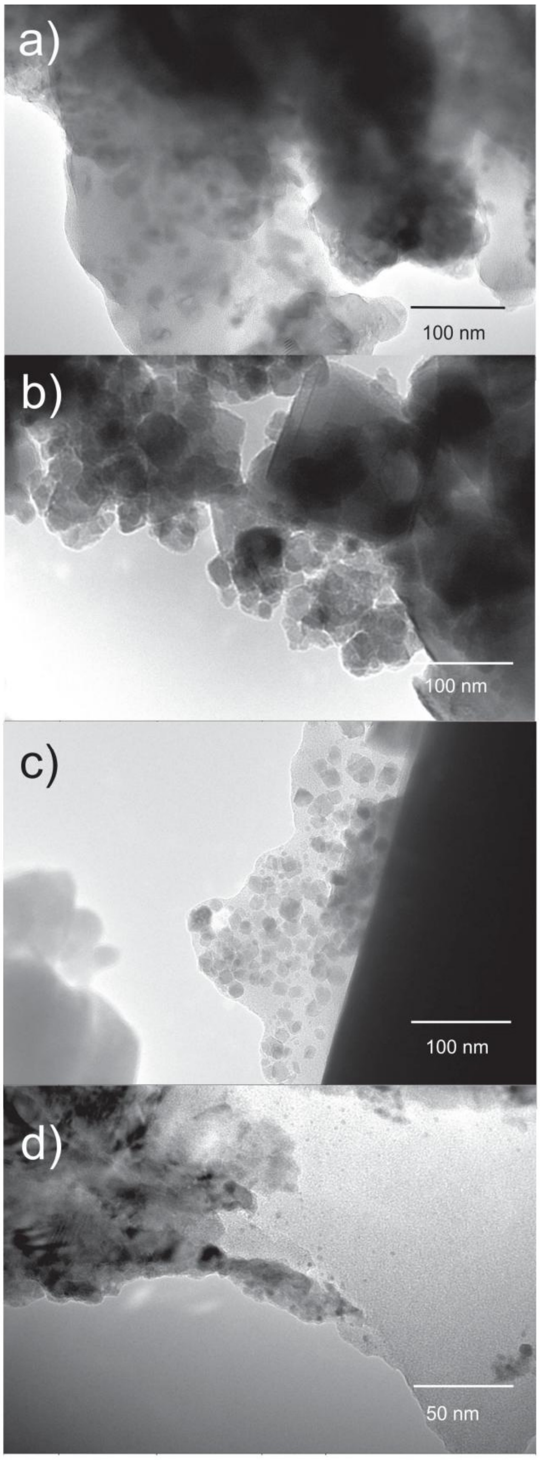

Figure S1. TEM micrographs a) CQD_25, b) CQD_50,c) CQD_100, and d) CQD_200 samples, respectively.

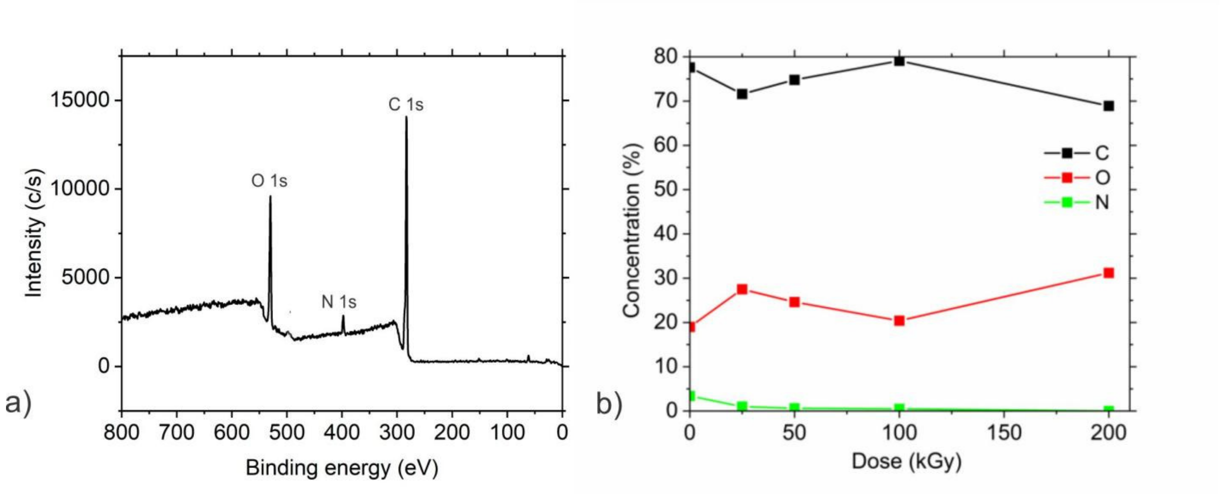

Figure S2. a) A typical survey XPS spectrum from CQDs_0 sample, b) Surface composition in at.% measured from the XPS spectra after gamma rays irradiation at different doses.

Table S1. The content in at.% of characteristic bonds identified in all investigated samples.

Dose (kGy)	C1 in C 1s	C2 in C1s	C3 in C1s	C4 in C1s	O1 in O 1s	O2 in O 1s
0	77	12	10	2	66	34
25	73	11	14	3	59	41
50	76	10	12	2	63	37
100	85	4	10	1	86	14
200	70	5	26	0	100	0

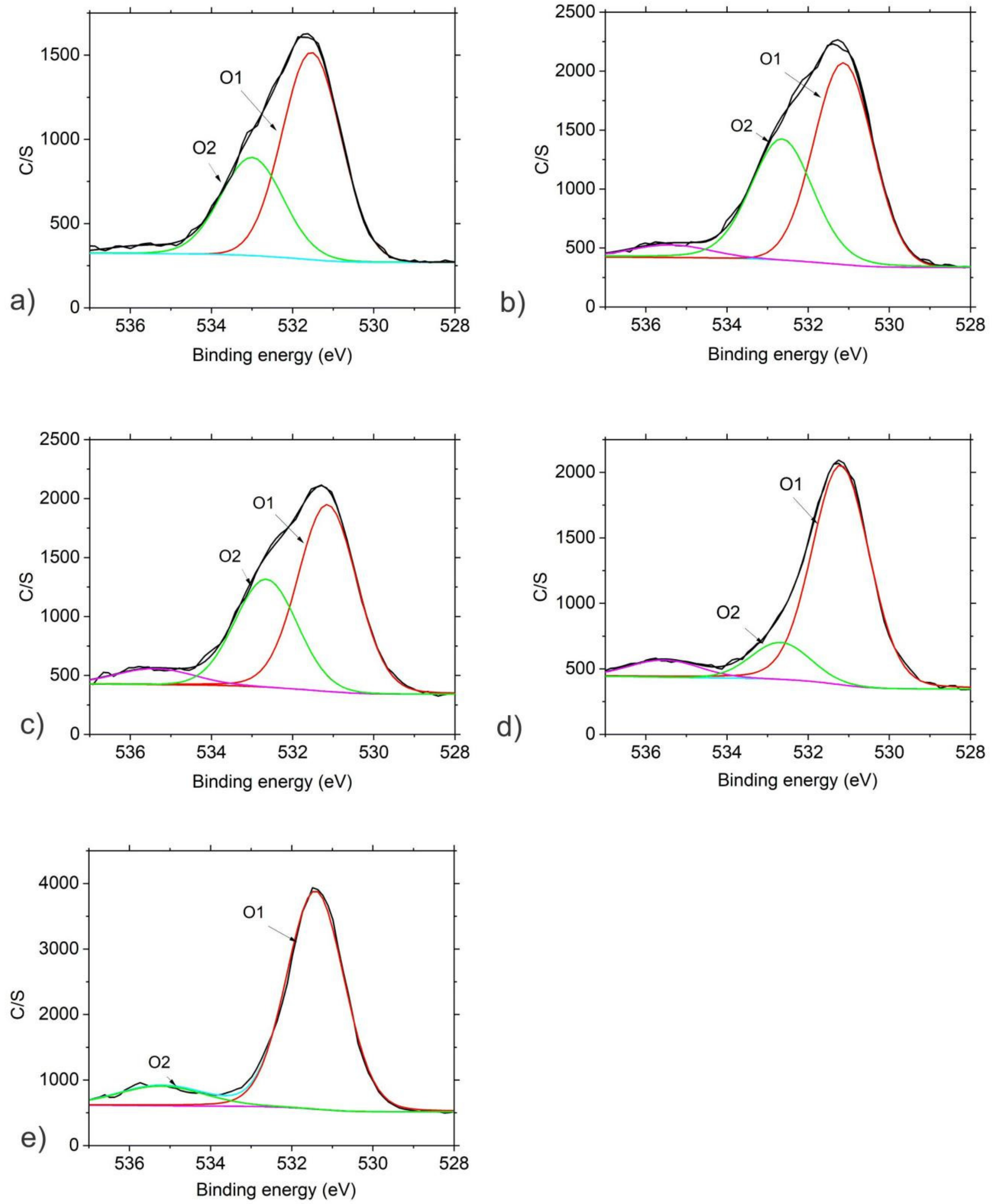

Figure S3. Fitted XPS O1s spectra of a) CQDs_0, b)CQDs_25, c) CQDs_50, d) CQDs_100, e) CQDs_200 samples, respectively.

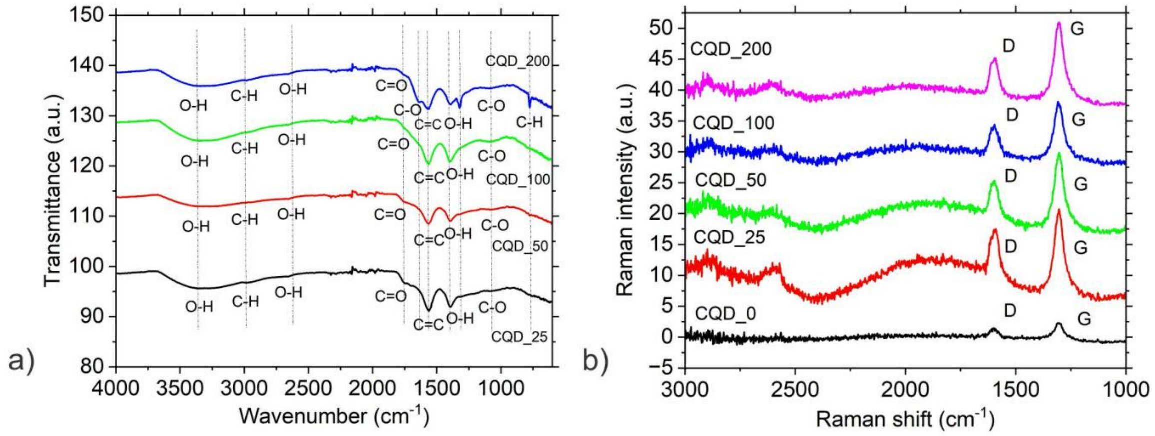

Figure S4. a) FTIR spectra of CQD_25, CQD_50, CQD_100, CQD_200, respectively, b) Raman spectra of CQD_0, CQD_25, CQD_50, CQD_100, CQD_200, respectively. All spectra are displaced due to clarity.

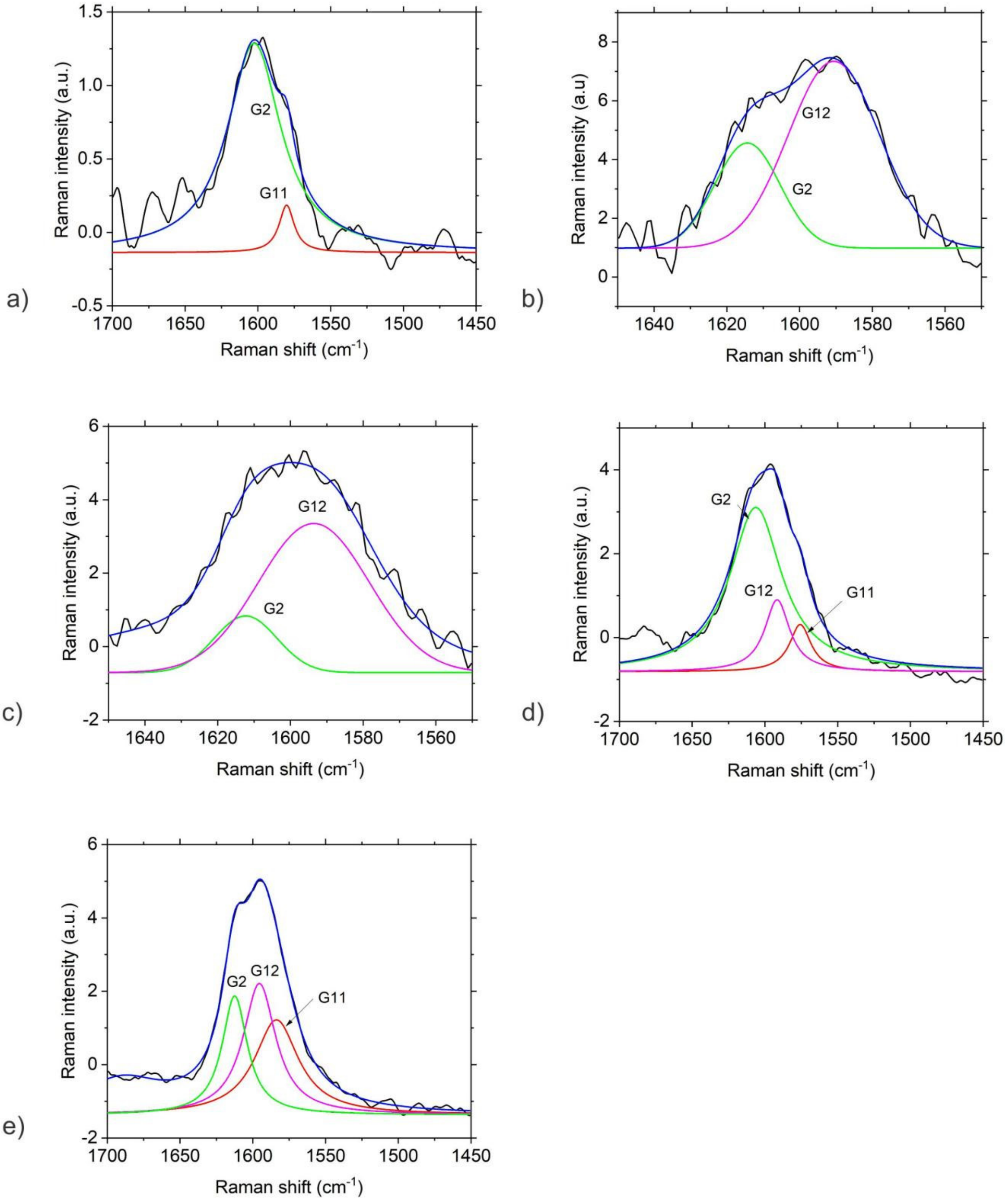

Figure S5. Fitted Raman spectra of a) CQD_0, b) CQD_25, c) CQD_50, d) CQD_100 and e)CQD_200. The G peaks of the samples were fitted by three Lorentzian peaks (1580, 1590 and 1610 cm^{-1}) which are denoted as G11, G12 and G2, respectively.

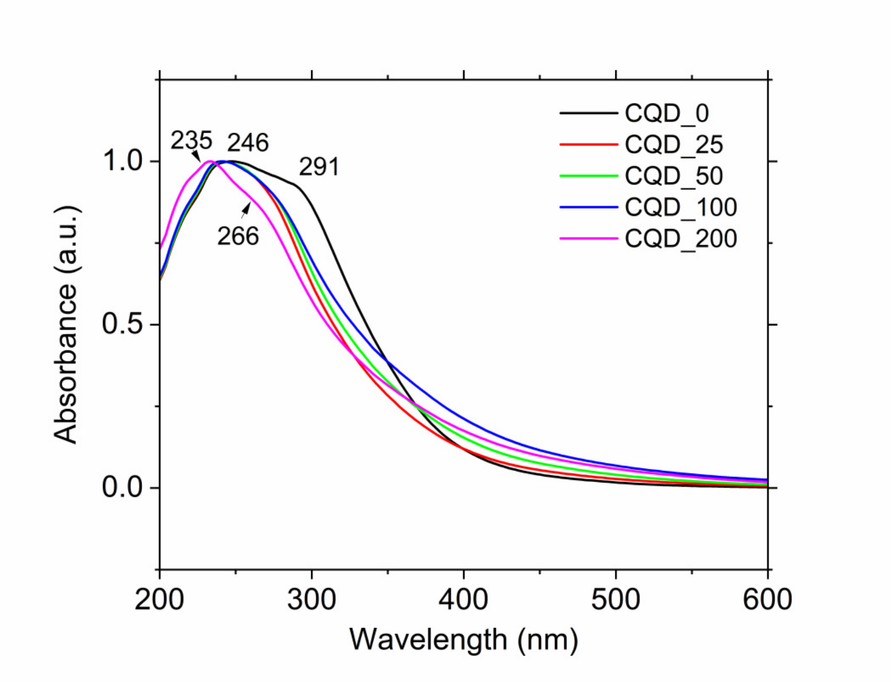

Figure S6. UV-Vis spectra of CQD_0 (black curve), CQD_25 (red curve), CQD_50 (green curve), CQD_100 (blue curve) and CQD_200 (magenta curve) samples, respectively.

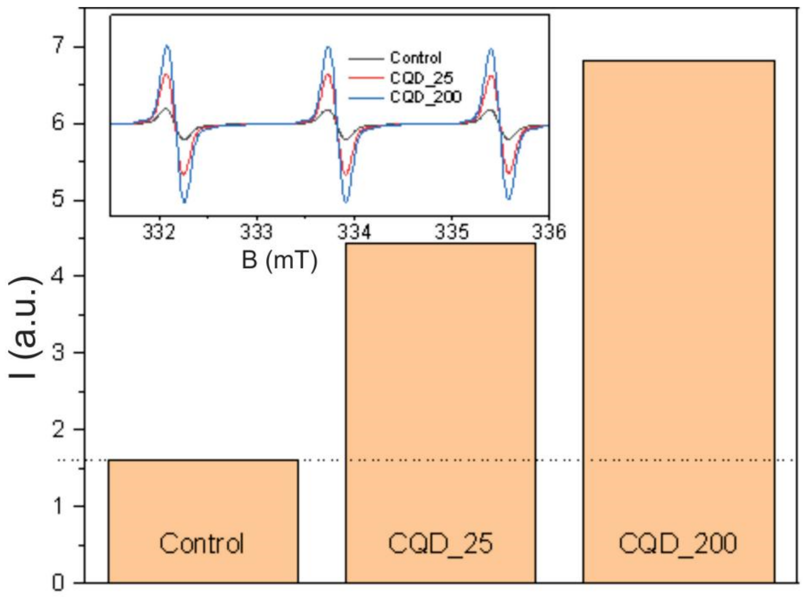

Figure S7. EPR intensity of singlet oxygen production by CQD_25 and CQD_200 samples compared to control.
